# Supplementary material for: Leveraging machine learning to unravel the impact of cadmium stress on goji berry micropropagation
Source: PLoS One. 2024 Jun 13;19(6):e0305111. doi: 10.1371/journal.pone.0305111 (PMC11175477; doi:10.1371/journal.pone.0305111)
Supplement: Attachment — Submitted filename: Response to Reviewers.docx [file pone.0305111.s003.docx]

Dear Editor,

Thank you again, the Editor and the reviewers, for your valuable comments on our manuscript. Below, please find our rebuttal to each peer's comment, including what edits were made to address them.

We hope these significant edits merit publication in PLOS ONE.

Sincerely,
Assoc. Prof. Dr. Özhan Şimşek

**Journal Requirements Response:**

1. **PLOS ONE Style Requirements:**

We have ensured that our manuscript meets PLOS ONE's style requirements, including file naming conventions.

2. **Code Sharing Guidelines:**

We have reviewed the PLOS ONE guidelines on code sharing for submissions where author-generated code underpins the findings in the manuscript. We have ensured that all author-generated code used in our study is available without restrictions upon publication. The code is provided as supplementary files.

3. **Data Availability Statement:**

Our current Data Availability Statement is as follows: "All relevant data are within the manuscript and its Supporting Information files."

We confirm that our submission contains all raw data required to replicate the results of our study. The minimal data set, including the values behind the means, standard deviations, and other measures reported, as well as the values used to build graphs and the points extracted from images for analysis, are included within the manuscript and its Supporting Information files.

We have also uploaded all raw data and code as supplementary files to ensure full compliance with the data availability requirements.

Thank you for your consideration.

**RESPONSES TO REVIEWER COMMENTS**

| Reviewer # 1 | |
| --- | --- |
| Comments | Reponses |
| While the study demonstrates a comprehensive approach to examining the effects of cadmium stress on Goji Berry micropropagation across multiple genotypes using various machine learning algorithms, it requires major revisions before publication. The paper lacks clarity regarding the methodology employed in the experiments, including the specific protocols for cadmium stress application and micropropagation techniques. | Thank you for your valuable comments on our manuscript. We appreciate your insights and have made the necessary revisions to address your concerns.  We have clarified the methodology section, providing detailed descriptions of the protocols used for cadmium stress application and the micropropagation techniques employed in our experiments. |
| Additionally, there is insufficient detail provided on how the machine learning models were trained and evaluated, hindering reproducibility and validity. | We have clarified the methodology section, providing detailed descriptions of the protocols used for cadmium stress application and the micropropagation techniques employed in our experiments. We have revised the methods section to include detailed information on how the machine learning models were trained and evaluated. This includes descriptions of the datasets used, the training process, the evaluation metrics, and any validation techniques applied. |
| Moreover, while the study identifies genotype-specific responses to cadmium stress, it fails to adequately discuss the underlying physiological mechanisms driving these responses, limiting the depth of interpretation. | We have expanded the discussion section to include an in-depth analysis of the underlying physiological mechanisms driving the genotype-specific responses to cadmium stress. This includes a detailed examination of relevant literature and our experimental findings to provide a more comprehensive interpretation of the results. |
| Furthermore, the practical implications of the findings for mitigating heavy metal stress in plants are mentioned but not sufficiently explored or substantiated with empirical evidence or real-world applications. | We have added sections to discuss the practical implications of our findings for mitigating heavy metal stress in plants. These additions include empirical evidence and potential real-world applications based on our results and related studies. |
| Overall, significant improvements in experimental design, methodology transparency, data interpretation, and practical relevance are necessary to enhance the rigor and impact of this study. | We have made significant improvements in experimental design, methodology transparency, data interpretation, and practical relevance. |
| The transition between discussing cadmium stress in plants and the application of machine learning in plant tissue culture is abrupt and lacks a clear connection. Providing a smoother transition or integrating these topics more seamlessly would enhance the flow of the introduction and help readers understand the relevance of each section to the overall study. | We have revised the introduction to provide a smoother transition between discussing cadmium stress in plants and the application of machine learning in plant tissue culture. These revisions aim to integrate the topics more seamlessly, enhancing the flow and helping readers understand the relevance of each section to the overall study. |
| While the introduction briefly mentions the application of machine learning in plant tissue culture, it does not provide sufficient context or justification for this approach. Explaining the potential benefits of using machine learning in the context of micropropagation studies and highlighting the limitations of traditional statistical methods would strengthen this aspect of the introduction. | We have revised the introduction to provide a more detailed explanation of the potential benefits of using machine learning in the context of micropropagation studies. This includes highlighting the limitations of traditional statistical methods. These improvements were made using the sources suggested by the reviewer. |
| The introduction should clearly outline the objectives and significance of the study. While it introduces various topics related to goji berry cultivation, cadmium stress, and machine learning, it is essential to explicitly state how these topics converge to address the research question or hypothesis of the paper. | We have revised the introduction to clearly outline the objectives and significance of the study. |
| The division of the dataset into training and testing subsets using a 10-fold cross-validation method is appropriate for evaluating predictive performance. However, additional details on how the dataset was partitioned, such as the ratio of training to testing data and any stratification methods employed, would strengthen the reproducibility and robustness of the analysis. | We have added detailed information on how the dataset was partitioned for the 10-fold cross-validation method. This includes the ratio of training to testing data and any stratification methods employed. |
| While the utilization of R programming and relevant packages (Caret and Kernlab) for implementing machine learning algorithms is suitable, providing a brief overview of the specific functions and parameters used within these packages would assist readers in understanding the modeling process.  It's essential to address any potential biases or limitations in the experimental design or modeling approach. For example, discussing any inherent variability in the dataset or potential confounding factors that may influence the outcomes would strengthen the credibility of the study. | We have added a brief overview of the specific functions and parameters used within the Caret and Kernlab packages in R for implementing the machine learning algorithms. This addition aims to assist readers in understanding the modeling process. We have addressed potential biases and limitations in the experimental design and modeling approach. This includes discussing any assumptions made, possible sources of bias, and how they were mitigated. |
| It is essential that the authors include standard deviation values for Tables 1, 2, 3, 4, and 5 to provide a measure of the variability within the data. This addition is crucial for assessing the reliability and consistency of the results presented in these tables. | We have included standard deviation values for Tables 1, 2, 3, 4, and 5 to provide a measure of the variability within the data. |
| In Table 6, the absence of performance criteria (R2, RMSE, and MAE) for both the training and testing sets diminishes the comprehensiveness of the analysis. Including these metrics for both sets would offer a more comprehensive evaluation of the predictive performance of the models. | We have revised Table 6 to include the performance criteria (R2, RMSE, and MAE) for both the training and testing sets. |
| The absence of images depicting the in vitro-grown plantlets and their responses to cadmium stress is a notable limitation in the study. Visual representations play a crucial role in elucidating experimental findings and enhancing the reader's understanding of the observed phenomena. Integrating images would provide valuable insights into the morphological changes and responses of the plantlets under different experimental conditions. | Thank you for your valuable feedback regarding the inclusion of images depicting the in vitro-grown plantlets and their responses to cadmium stress. We fully understand and agree that visual representations play a crucial role in elucidating experimental findings and enhancing the reader's understanding of the observed phenomena. Unfortunately, we regret to inform you that the images taken during the study were stored on a specific memory card. Due to the unfortunate circumstances surrounding the major earthquake disaster in Turkey on February 6, 2023, there was a period of significant confusion and disruption. During this time, our laboratory remained uncontrolled for an extended period, and the memory card containing these important images was lost and has not been recovered. We are deeply saddened by this loss, as we recognize the importance of these images for our study. Despite this, we have ensured that all raw data and codes are included as supplementary files to provide as much transparency and detail as possible. We sincerely apologize for this limitation and appreciate your understanding given these extraordinary circumstances. We hope that the comprehensive data and analysis we have provided will still meet the rigorous standards required for publication. |

| Reviewer # 2 | |
| --- | --- |
| Comments | Reponses |
| More details on the motivations and experimental results should be clarified in the Abstract and Introduction Section. | We have revised the Abstract and Introduction sections to provide more detailed information on the motivations behind our study and to clarify our experimental results. These revisions aim to offer a clearer context and a more comprehensive summary of our findings. |
| To make the reference list cover more related works and improve the readability of this manuscript, I suggest that authors refer to the following works, if available:  1.Machine learning-based prediction of lymph node metastasis among osteosarcoma patients.  2.Development of a Machine Learning-Based Predictive Model for Lung Metastasis in Patients with Ewing Sarcoma. | We appreciate the suggestion to include more related works to improve the readability and coverage of our reference list. We have reviewed and referred to the following works in our manuscript. |
| More comparisons with SOTA works should be included to verify the efficiency of the proposed work. | We have included additional comparisons with state-of-the-art (SOTA) works to verify the efficiency of our proposed methods. These comparisons are discussed in the Results and Discussion sections, highlighting how our approach stands relative to the current leading methodologies in the field. |
| Please clarify the reason that using proposed methods in this work. Why not some other novel machine learning methods? | The choice of machine learning methods in this study, including Multilayer Perceptron (MLP), Support Vector Machines (SVM), Random Forest (RF), Gaussian Process (GP), and Extreme Gradient Boosting (XGBoost), was driven by several key factors. Each of these methods has distinct strengths that are particularly suited to the complex and high-dimensional nature of our dataset. Traditional statistical methods often struggle to analyze such intricate datasets effectively.   - **Multilayer Perceptron (MLP)** and **Support Vector Machines (SVM)** are powerful tools for capturing non-linear relationships, which are common in biological data. These models excel in their ability to model complex interactions between variables. - **Random Forest (RF)** and **Extreme Gradient Boosting (XGBoost)** are ensemble learning methods known for their robustness and ability to handle large amounts of data with many features. These methods are less prone to overfitting and provide reliable performance across diverse datasets. - **Gaussian Process (GP)** offers a probabilistic approach to learning, providing not only predictions but also a measure of uncertainty, which is crucial for understanding the confidence in our model's predictions.   While novel machine learning methods continue to emerge, our choice focused on these well-established models due to their proven effectiveness in various domains, including plant science, as corroborated by numerous studies. This selection ensures that our results are robust, reliable, and comparable with existing literature.  By leveraging these methods, we aimed to harness their individual and collective strengths to achieve a comprehensive and accurate analysis of the relationships between cadmium stress and micropropagation in goji berries. This approach enhances the interpretability and practical applicability of our findings, contributing valuable insights into developing strategies to mitigate heavy metal stress in plants. |
| Please present more comparisons with currents machine learning works. | We have included more comparisons with current machine learning works to verify the efficiency of our proposed methods. These comparisons are detailed in the Results and Discussion sections, highlighting how our approach stands relative to recent advancements in the field. |
| Please also describe the strengthen and weakness of current works and the main contributions of the proposed framework. | We have described the strengths and weaknesses of current works and detailed the main contributions of our proposed framework. These additions are included in the Discussion section, providing a balanced view of how our work builds on and improves upon existing methodologies. |
| More conclusions and discussions on future works need to be included. | We have expanded the Conclusions and Discussion sections to include more detailed conclusions and discussions on future works. |
